# Chemical and Cellular Antioxidant Activities of In Vitro Digesta of Tilapia Protein and Its Hydrolysates

**DOI:** 10.3390/foods9060833

**Published:** 2020-06-25

**Authors:** Xiaogang Zhang, Parinya Noisa, Jirawat Yongsawatdigul

**Affiliations:** 1 Institute of Agricultural Technology, School of Food Technology, Suranaree University of Technology, 111 University Avenue, Nakhon Ratchasima 30000, Thailand; zxg-food@hotmail.com; 2 Institute of Agricultural Technology, School of Biotechnology, Suranaree University of Technology, 111 University Avenue, Nakhon Ratchasima 30000, Thailand; p.noisa@sut.ac.th

**Keywords:** antioxidant activity, protein hydrolysate, in vitro GI digestion, oxidative stress

## Abstract

Production of protein hydrolysate as nutraceuticals is typically based on the activity of the hydrolysate, which might not yield the optimal activity under physiological condition due to structural modification of peptides upon gastrointestinal (GI) digestion. This study systematically compared the chemical and cellular antioxidant activities of the in vitro digesta of tilapia protein and its hydrolysates prepared with various degree of hydrolysis (DH) by Alcalase. The enzymes used in the in vitro GI digestion analysis significantly contributed to the peptide content, Trolox equivalent antioxidant capacity (TEAC), and oxygen radical absorbance capacity (ORAC). Proteins and all hydrolysates were slightly digested by pepsin but hydrolyzed extensively by pancreatin. Both hydrolysate and digesta predominantly scavenged free radicals via hydrogen atom transfer (HAT). The antioxidant activities of the hydrolysates increased with the increasing DH up to 16 h of hydrolysis. However, the digesta of 10-h hydrolysate displayed the highest chemical and HepG2 cellular antioxidant activities, while the protein digesta displayed the lowest. Principal component analysis (PCA) showed that the TEAC of the digesta was positively correlated with the cellular antioxidant activity (CAA). Therefore, the production of protein hydrolysate should be optimized based on the activity of the hydrolysate digesta rather than that of hydrolysates.

## 1. Introduction

Antioxidant peptides derived from food proteins have been widely elucidated and characterized in various food protein sources [1,2,3,4]. They have been considered as a natural antioxidant, which can retard oxidative deterioration of food during storage [4,5,6]. In addition, antioxidant peptides have been shown to protect biological systems from oxidative damages which can cause many pathophysiological processes such as aging, inflammation, diabetes, and cancer [4,7,8]. Inclusion of the protein hydrolysate containing antioxidant peptides in feeds has also ensured desirable rates of growth performance and feed efficiency in pigs, calves, poultry, and fish [9,10,11]. The underlying mechanisms are related to improvement in intrinsic total antioxidant capacity, immune response, among others. However, when protein hydrolysates are applied as functional foods or an ingredient in functional feed, the oral administration likely modifies their activities due to hydrolysis occurring during gastrointestinal (GI) digestion by proteases [12,13]. In vitro GI digestion increased the antioxidant activities of hydrolysates derived from fish muscle protein or skin [3,14,15]. Nevertheless, the antioxidant activities of the positively charged fraction of casein hydrolysate and synthesized peptide, ATSHH, decreased after digestion [12,16]. Therefore, the antioxidant activities of protein hydrolysate might not totally correlate with those of the digesta. Thus, the process optimization of protein hydrolysate, which is based merely on the antioxidant activities of the hydrolysate, might not yield the product with the optimal health benefits. However, this approach has been commonly practiced for the production of protein hydrolysate with bioactivities [17,18,19]. The antioxidant activities of the digesta would be more physiologically relevant and should be considered as one of the criteria for optimizing production of the antioxidant hydrolysate. Comparison between activities of the hydrolysate and its digesta has not been systematically elucidated.

Evaluation of food digestion in humans is the most reliable technique, but the trials are difficult to control, expensive, and might not all be justifiable on ethical grounds. Therefore, the static or dynamic in vitro models have been used for many decades to simulate the digestion of food [20,21,22]. Dynamic models provide more physiologically relevant data than static counterparts, but they are more time-consuming, expensive, and difficult to access [21,22]. A standardized static in vitro digestion method has been established by the COST action INFOGEST [20,23]. Egger et al. [24] evaluated digestion of milk protein by the INFOGEST static method and found that the endpoint of digestion was comparable to in vivo protein hydrolysis. Therefore, the in vitro static method has been accepted as a useful technique to assess the endpoints of food digestion.

The digesta of protein hydrolysate contained peptides and amino acids that resulted from gastric (pepsin) and intestinal (pancreatin) digestion. Nevertheless, peptides/amino acids derived from the proteases used in the analysis could also be generated due to the autolysis and/or hydrolysis [25,26]. This could also contribute to the activities of interest. Dave et al. [25] reported that peptides derived from trypsin by in vitro digestion revealed the 2,2′-azino-bis(3-ethylbenzothiazoline-6-sulfonic acid) diammonium salt radical cation (${\mathrm{ABTS}}^{\text{•}+}$) and *α*, *α*-diphenyl-*β*-picrylhydrazyl free radical (DPPH•) scavenging capacity. Therefore, the antioxidant activities of the peptides derived from the enzyme blanks of in vitro model should be assessed to obtain more biologically relevant antioxidant activity of the digesta. Thus far, the issue of enzyme blanks has not been well characterized in publications.

Many chemical assays with different mechanisms have been conducted to evaluate the antioxidant activity of the interested compounds [4,27]. Combining chemical assays under various mechanisms could be useful to interpret the primary mechanisms of the antioxidant peptides. The cellular antioxidant activity (CAA) assay is a more biologically relevant technique, as it addresses some issues of cells, such as their uptake, distribution, and ability to metabolize the antioxidant [27,28]. If chemical antioxidant assays could be used to predict the biological activity of peptides, it would greatly facilitate the optimization of antioxidant hydrolysates/peptides production with more relevant health benefits. However, the relationship between the chemical and cellular antioxidant activities of food peptides has not been well established.

To increase the value and utilization of tilapia, protein hydrolysates with antioxidant activities should be produced under the optimal condition. The objectives of this study were to assess antioxidant activity of the in vitro GI digesta of tilapia hydrolysate with various degrees of hydrolysis (DH). Cytoprotection and intracellular ROS scavenging capacity assays based on HepG2 cell lines were also carried out. The relationships between the chemical and cellular antioxidant activities were also established based on principal component analysis (PCA) and Pearson correlation analysis.

## 2. Materials and Methods

### 2.1. Chemicals and Reagents

Alcalase^®^ 2.4 L FG was purchased from Novozymes (Bagsvaerd, Denmark). Pepsin (from porcine gastric mucosa), pancreatin (from porcine pancreas), 3-morpholinosydnonimine hydrochloride (SIN-1), dihydrorhodamine 123 (DHR 123, ≥95.0%), fluorescein sodium salt (FL, BioReagent), 2,4,6-trinitrobenzenesulfonic acid solution (TNBS, 5.0%, w/v, BioReagent), reduced L-glutathione (GSH, ≥98.0%), cytochrome C, aprotinin, hippuryl-His-Leu acetate salt (Hip-His-Leu), 2,2′-azinobis(3-ethylbenzothiazoline-6-sulfonic acid) diammonium salt (ABTS, ≥98.0%), 3-(2-pyridyl)-5,6-diphenyl-1,2,4-triazine-*p*,*p*′-disulfonic acid monosodium salt hydrate (ferrozine, 97.0%), (±)-6-hydroxy-2,5,7,8-tetramethylchromane-2-carboxylic acid (Trolox, 97.0%), 2,4,6-tris(2-pyridyl)-*s*-triazine (TPTZ, ≥99.0%), 3-(4,5-dimethyl-2-thiazolyl)-2,5-diphenyl-2H-tetrazolium bromide (MTT), and 2′7′-dichlorodihydro-fluorescein diacetate (DCFH-DA) were purchased from Sigma-Aldrich (St. Louis, MO, USA). 2,2′-Azobis (2-methylpropionamidine) dihydrochloride (AAPH, 98.0%) and tryptophan (99.0%) were bought from ACROS Organics™ (Morris Plains, NJ, USA). *L*(+)-ascorbic acid was obtained from CARLO ERBA Reagents S.A.S (Rodano, Italy). The synthetic peptides, AGNQVLNLQADLPK (AK-14) and NTFLFFK (NK-7), were obtained from GL Biochem (Shanghai) Ltd. (Shanghai, China). Dulbecco’s modified eagle medium (DMEM) and fetal bovine serum (FBS) were purchased from HyClone (HyClone, Logan, UT, USA). Trypsin-ethylenediaminetetraacetic acid (EDTA), *L*-Gln, and non-essential amino acids (NEAAs) were obtained from Gibco (Carlsbad, CA, USA) for the cell culture studies. Acetonitrile (ACN) and trifluoroacetic acid (TFA) were of HPLC grade. All the other chemicals were of analytical grade.

### 2.2. Preparation of Protein Hydrolysates

Fresh tilapia (*Oreochromis niloticus*) was bought from a local market in Nakhon Ratchasima, Thailand. Fish were stored in ice and transported to the laboratory at Suranaree University of Technology within 1 h of their purchase. Fish were descaled, gutted, and skinned. Dorsal meat was collected and minced using a mincer (Kenwood A920, Havant, UK). The tilapia mince was defatted two times using isopropanol at a sample to solvent ratio of 1:4 (w/v) at 40 °C for 20 min. Subsequently, the defatted sample was left in the fume hood and dried in a hot air oven at 60 °C for 3 h. The sample was ground and passed through a 230-mesh sieve to obtain tilapia protein powder (*P*), which was vacuum-packed and stored at −18 °C for further use. The proximate composition of *P* was analyzed according to AOAC [29].

*P* containing 2 g of protein was suspended in 20 mL of 0.15 M NaHCO_3_ to reach a final protein content of 10% (w/v), and the pH was then adjusted to 8.0 ± 0.1 using 10 M NaOH. The protein was hydrolyzed by 5% Alcalase (w/w of protein) in a 160-rpm shaking water bath at 50 °C for various time intervals of 2, 6, 10, and 16 h. Subsequently, the samples were heated at 90 °C for 10 min to terminate the enzyme activity and cooled immediately in ice water. The pH of the hydrolysate was adjusted to 7.0 ± 0.1, and the volume was brought to 25 mL. The samples were then centrifuged at 10,000× *g* and 4 °C for 20 min, filtered through Whatman 4 filter paper, and denoted as *H*_2_, *H*_6_, *H*_10_, and *H*_16_ for hydrolysis times of 2, 6, 10, and 16 h, respectively. The hydrolysates were immediately subjected to simulated in vitro GI digestion, as described in 2.3.

Thermally inactivated Alcalase (*A*_0_) was also prepared by heating Alcalase at 90 °C for 10 min. The heated-*P* with inactivated Alcalase, denoted as *P*_0_-*A*_0_, was prepared by adding thermally inactivated Alcalase into the *P*, which was previously heated at 90 °C for 2 min and further heated for 8 min.

### 2.3. Simulated In Vitro GI Digestion

Simulated in vitro GI digestion was carried out according to Minekus et al. [23] with slight modifications. The oral phase, *α*-amylase and lipase were ignored as tilapia protein powder and liquid hydrolysates contained mainly proteins/peptides. Gastric digestion was prepared by mixing 5 mL of hydrolysate, 3.2 mL of the simulated gastric fluid (*SGF*) stock solution, and 0.075 mM CaCl_2_ in the final mixture. The mixture was adjusted to a pH of 3.0 ± 0.1 using 6 M HCl and maintained a total volume of 9.2 mL. After pre-incubation in a 37 °C water bath for 15 min, freshly prepared pepsin in an *SGF* stock (0.8 mL) was added to achieve a 2000 U/mL in the final mixture. The mixture was digested for 2 h at 37 °C with 160-rpm shaking. For intestinal digestion, 5.5 mL of the simulated intestinal fluid (*SIF*) stock solution and 0.20 mL of 10 M NaOH were rapidly mixed with 10 mL of gastric chime. The pH was adjusted to a pH ˃ 5.6, and CaCl_2_ was added to attain a 0.3 mM concentration in the final mixture. The mixture was finally adjusted to a pH of 7.0 ± 0.1 and a total volume of 17.5 mL. After pre-incubation in a 37 °C water-bath for 15 min, freshly prepared pancreatin in an 2.5 mL of *SIF* stock was added to achieve a 100 U/mL, which was determined based on the trypsin activity of the final mixture, and the mixture was digested for a further 2 h at 37 °C.

To terminate the enzyme in either the gastric (*GD*) or gastric-intestinal digesta (*GID*), the samples were heated at 90 °C for 10 min and cooled immediately in ice water. The *GD*s of the hydrolysates were expressed as *H*_2_-*GD*, *H*_6_-*GD*, *H*_10_-*GD*, and *H*_16_-*GD*, while the *GID*s were referred to as *H*_2_-*GID*, *H*_6_-*GID*, *H*_10_-*GID*, and *H*_16_-*GID* for hydrolysates obtained with 2, 6, 10, and 16 h of hydrolysis, respectively. The *GD*s and *GID*s were directly used for the chemical assays. The *GID*s were also lyophilized for the cellular assays.

The protein digesta were also prepared using the same amount of the initial protein and expressed as *P*-*GD* and *P*-*GID* for the gastric and GI digesta, respectively. Several enzyme blanks were prepared during digestion and expressed as thermally inactivated pepsin (*Pep*_0_), inactivated pancreatin (*Pan*_0_), and an enzyme blank of GI digestion (*Pep*-*Pan*-I), which was prepared with the procedure described for the in vitro GI digestion, but without peptide samples. The contribution of Alcalase (*A*_0_) on antioxidant activity was also investigated by incubating the inactivated *A*_0_ following the in vitro GI digestion and was denoted as *A*_0_-*GID*.

### 2.4. DH and Digestibility

The *α*-amino group content was analyzed by a TNBS assay [30] with slight modifications. A sample diluted in 50 μL of 1% SDS was added 250 μL of 0.2125 M phosphate buffer (pH 8.2) and 250 μL of freshly diluted 0.05% TNBS, and incubated at 50 °C for 1 h in the dark. Then, 500 μL of 0.1 M HCl was added to stop the reaction. After incubation at room temperature for 30 min, the absorbance was measured absorbance at 420 nm (Stone, Staffordshire, ST15 OSA, UK). *L*-Leu was used as a standard. The total *α*-amino group content of the sample was measured by hydrolyzing the sample in 6 M HCl at 110 °C for 24 h in a heating block (Boekel Scientific, Feasterville, PA, USA). Subsequently, the pH was adjusted to 7.0 ± 0.1 for the TNBS analysis. The DH was calculated using Equation (1) according to Benjakul and Morrissey [31]. The digestibility was calculated using Equation (2).

(1) $$\mathrm{DH}\;(\%)=(\frac{L_{t}-L_{0}}{L_{\mathrm{total},p}-L_{0}})\times 100,$$

(2) $$\mathrm{Digestibility}\;(\%)=\left(\frac{L_{\mathrm{ad}}{\;-\;L}_{\mathrm{bd}}}{L_{\mathrm{total},h}{\;-\;L}_{\mathrm{bd}}}\right)\text{ × }100,$$

where, L_t_, L_0_, and L_total,p_ were the *α*-amino group contents of hydrolysate at time t and 0 h and the total *α*-amino group of protein, respectively. L_bd_ and L_ad_ were the *α*-amino group content before and after in vitro digestion, respectively. L_total,h_ was total *α*-amino group content of the respective hydrolysate. For protein digestibility, L_total,p_ was used instead of L_total,h_. The results were calculated after the subtraction of the respective enzyme blanks.

### 2.5. Molecular Weight (MW) Distribution

The samples were diluted with the mobile phase (7:3 v/v of 0.1% TFA: ACN), and 100 μL was injected into a Superdex Peptide 10/300 GL column equipped with fast protein liquid chromatography (FPLC) system (ÄKTA purifier, GE Healthcare Bioscience Co., Uppsala, Sweden). The peptides were eluted by the mobile phase at room temperature with a flow rate of 1.0 mL/min and total elution volume of 27 mL, which ensured complete elution of all peptides and column equilibration for the new run. Aprotinin (6512 Da), AK-14 (1481 Da), NK-7 (916 Da), Hip-His-Leu (429 Da), and tryptophan (204 Da) were used as the external standards. Cytochrome C (12,400 Da) was used to determine the void volume (*V*_0_). LogMW versus *K*_av_ of the standards was plotted, where *K*_av_ was defined according to Wu et al. [32].

### 2.6. Chemical Antioxidant Activity

#### 2.6.1. Trolox Equivalent Antioxidant Capacity (TEAC)

The TEAC assay was carried out according to the ABTS radical cation decolorization method [33] with slight modifications. The ${\mathrm{ABTS}}^{\text{•}+}$ stock containing 7 mM ABTS and 2.45 mM potassium persulfate was prepared and incubated in the dark for 12–16 h before use. Phosphate buffered saline (PBS, 10 mM, pH 7.4) containing 0.138 M NaCl and 0.0027 M KCl were used to the dilute ${\mathrm{ABTS}}^{\text{•}+}$ stock to obtain a working solution with an absorbance of approximately 0.7 at 734 nm. A 50 μL of sample was mixed with 950 μL ${\mathrm{ABTS}}^{\text{•}+}$ and incubated at room temperature for 15 min, and the absorbance was measured within 30 min. Trolox was used as a standard, and the activity was expressed as trolox equivalents (TE)/g initial protein.

#### 2.6.2. Ferric Reducing Antioxidant Power (FRAP)

The FRAP assay was carried out according to Benzie and Strain [34] with some modifications. The FRAP reagent was freshly prepared by mixing 300 mM acetate buffer at a pH of 3.6, 10 mM TPTZ in 40 mM HCl, and 20 mM FeCl_3_·6H_2_O at ratio of 10:1:1 (v/v), and incubated at 37 °C for 10 min. A 100 μL of sample was incubated with 1000 μL of the FRAP reagent at 37 °C for 10 min in the dark, and the absorbance was measured at 593 nm. The activity was expressed as TE/g initial protein.

#### 2.6.3. Fe^2^^+^ Chelating Capacity (FICC)

The FICC was assessed according to Decker and Welch [35] with slight modifications. A 50 μL of sample, 1000 μL of deionized water, and 50 μL of 1 mM FeCl_2_ were mixed and incubated for 5 min in the dark, and then, 50 μL of 5 mM ferrozine was added and incubated for a further 20 min. The absorbance was measured at 562 nm. EDTA was applied as a standard, and the results were expressed as EDTA equivalents/g initial protein.

#### 2.6.4. Oxygen Radical Absorbance Capacity (ORAC)

The ORAC assay was performed in a black 96-well microplate as described by Dávalos et al. [36] with slight modifications. Equal volumes (50 μL) of the sample, 150 mM PB (pH 7.4), and 280 nM fluorescein (FL) in 75 mM PB (pH 7.4) were mixed and incubated at 37 °C for 15 min. Subsequently, 50 μL of 48 mM AAPH in 75 mM PB was added, and the fluorescence at *λ*_Ex__/Em_ = 485/520 nm was recorded at 1-min intervals for 110 min (Varioskan LUX Multimode Microplate Reader, Thermo Fisher Scientific, Waltham, MA, USA). Blanks were prepared using DI water instead of the sample. The area under the curve (AUC) was estimated by subtracting the baseline using SkanIt^TM^ software 4.1 (Thermo Fisher Scientific, Waltham, MA, USA). The activity was expressed as TE/g initial protein.

#### 2.6.5. Peroxynitrite (${\mathrm{ONOO}}^{-}$) Scavenging

The peroxynitrite scavenging ability was evaluated according to Kooy et al. [37] with some modifications. The SIN-1 and DHR 123 stock solutions were prepared in a 50 mM cold deoxygenated PBS solution (pH 7.4) and freshly diluted by 50 mM PBS for further use. Equal volumes (50 μL) of the sample, 100 mM PBS (pH 7.4), 10 μM DHR 123, and 40 μM SIN-1 were placed in a 96-well black microplate at 37 °C, and the fluorescence at *λ*_Ex__/Em_ = 500/536 nm was recorded at 2.5-min intervals for 120 min. The blanks were prepared using DI water. After the baseline was subtracted, the integrated area of the fluorescence curve plotted as a function of time was calculated using SkanIt^TM^ Software 4.1. The results were expressed as GSH equivalents/g initial protein.

### 2.7. Cellular Antioxidant Activities

#### 2.7.1. Cell Culture

The human hepatocellular carcinoma (HepG2) cells were purchased from the American Type Culture Collection (ATCC, Manassas, VA, USA) and cultured in a humidified incubator at 37 °C with 5% CO_2_. The culture medium was DMEM containing 10% (v/v) heat-inactivated FBS, 1% (v/v) *L*-Gln, 1% (v/v) NEAAs, and 1% (v/v) penicillin-streptomycin (10,000 μg/mL). The growth medium was replaced by fresh media once every other day. After the HepG2 cells reached 80%–90% confluence in the Corning 75-cm^2^ culture flask, the cells were trypsinized using 0.05% trypsin-EDTA.

#### 2.7.2. Cytotoxicity and Cytoprotection

The cell seeding density was 2 × 10^4^ cells/well in a clear 96-well microplate. The cells were incubated and treated for 48 h as follows. The cell viabilities were detected by an MTT assay to evaluate the cytotoxicity and/or cytoprotection. The cytotoxicity of the digesta was detected by incubating the 24-h cultured cells with different concentrations (0–15 mg/mL) of the digesta for 24 h. The 50% lethality concentration of AAPH was determined for the cytoprotection assay by incubating the 36-h cultured cells with various concentrations of AAPH (0–60 mM) for 12 h. The cytoprotection of the digesta was measured by incubating the 24-h cultured cells with the digesta (0.1 and 2.0 mg/mL) for 12 h. Subsequently, 40 mM AAPH was used to induce cellular oxidative stress for 12 h. The non-treated cells, cells treated by AAPH only (oxidative stress control), and 0.05 mg/mL ascorbic acid positive control were also tested in the same plate as the plate containing the digesta samples. For the MTT assay, the MTT reagent prepared with a concentration of 0.5 mg/mL in 1 × PBS was incubated with the cells for 2 h in the dark. DMSO was used to dissolve formazan, and the absorbance was detected at 570 nm by a SPECTROstar NANO system (BMG LABTECH GmbH, Ortenberg, Germany).

#### 2.7.3. Intracellular ROS Scavenging Capacity

The intracellular ROS scavenging capacity was assessed as described by Wolfe et al. [28] with slight modifications. The cells were seeded to achieve 6 × 10^4^ cells/well and cultured on a sterile Corning^®^ 96-well black polystyrene microplate with a flat clear bottom until 100% confluence was reached. The digesta and 25 μM DCFH-DA prepared in the medium without FBS were added to the microplate and incubated for 1 h. Subsequently, the medium was discarded, the cells were washed once with 1 × PBS, and 600 µM AAPH in 1 × PBS was applied. After 1 h of incubation, the fluorescence intensity at *λ*_Ex__/Em_ = 485/538 nm was read at 37 °C. The cells treated by DCFH-DA only (cell blank), DCFH-DA followed by APPH (oxidative stress control), and 0.05 mg/mL ascorbic acid (positive control) were also studied in each plate along with the digesta samples. The results were expressed as the CAA unit and calculated according to Kellett et al. [38] using the following Equation (3).

(3) $$\mathrm{CAA}\;\mathrm{unit}=\left(1\;-\;\frac{{\mathrm{Fluorescence}\;\mathrm{intensity}}_{\mathrm{sample}}}{{\mathrm{Fluorescence}\;\mathrm{intensity}}_{\mathrm{oxidative}\;\mathrm{stress}\;\mathrm{control}}}\right)\text{ × }100$$

### 2.8. Statistical Analyses

All the tests were carried out in three replicates and expressed as the mean ± SD. Comparisons between the two means were performed using independent sample *t*-tests (*p* = 0.05). When there were more than two treatments, the mean differences were analyzed by ANOVA using Tukey HSD (IBM SPSS Statistics for Windows, version 23.0. Armonk, NY: IBM Corp) (*p* = 0.05). All the calculations, PCA, and Pearson correlation analysis were conducted with OriginPro 2018 (OriginLab Corporation, Northampton, MA, USA).

## 3. Results and Discussion

### 3.1. Antioxidant Activities of the Enzymes Used in Hydrolysis and In Vitro GI Digestion

The enzyme blanks of the hydrolytic reaction (Alcalase, *A*_0_) and gastric digestion (pepsin, *Pep*-I) displayed a negligible *α*-amino group content and antioxidant activities compared with those of the digesta of 10-h hydrolysate (*H*_10_-*GID*^#^, Table 1). However, when pepsin and pancreatin were prepared as the enzyme blank for the GI digestion process (*Pep*-*Pan*-I), the *α*-amino content and antioxidant activities (TEAC and ORAC) were approximately 15%–40% of those of *H*_10_-*GID*^#^. These results suggested that the peptides derived from pancreatin possessed significant levels of *α*-amino and antioxidant activities. These peptides are probably generated by the autolysis and/or hydrolysis of the digestive enzymes presented [25,26]. Moreover, some peptides might have originally been present in the commercial enzymes, as seen from the MW distribution of the thermally inactivated pancreatin (*Pan*_0_) (Table 2). Furthermore, *A*_0_ could be digested to antioxidant peptides, resulting in *A*_0_-*GID* having higher activities than *Pep*-*Pan*-I (Table 1). Therefore, the *α*-amino content and antioxidant activities of the enzyme blanks indicated that the proteases used in the hydrolysate and in vitro GI digestion appeared to significantly contribute to the *α*-amino content and antioxidant activities observed in the digesta. Nevertheless, most published studies disregarded or at least did not clarify the contribution of the peptides derived from the digestive enzymes. The antioxidant activities of the hydrolysates derived from the fish muscle protein, skin, or surimi byproduct were increased by in vitro digestion [1,3,14,15,39]. However, no significant alteration in the antioxidant activities of casein hydrolysate were observed after digestion [16]. Pancreatin digestion decreased the ORAC of the dipeptide SM, and TEAC of tripeptide NCS [40]. Our results revealed that the enzyme blanks prepared for the standard in vitro GI analysis are necessary, and readings from blank should be subtracted from the sample to reflect the true antioxidant activity value of the hydrolysate/protein digesta.

### 3.2. DH and MW Distribution

**Figure 1:**
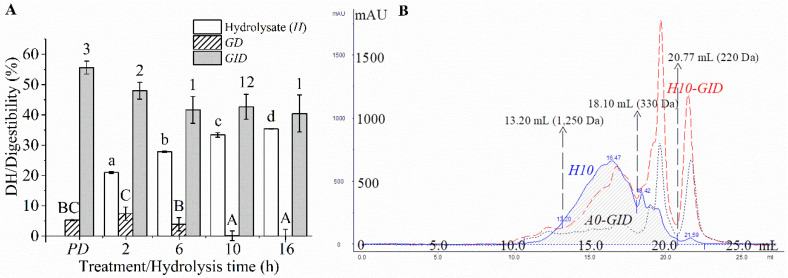
Degree of hydrolysis (DH) or digestibility of the protein or its hydrolysates (**A**) and size exclusion chromatograms (SECs) of the enzyme blank, which was the digesta of thermally inactivated Alcalase (*A*_0_-*GID*), 10-hydrolysate (*H*_10_), and its digesta (*H*_10_-*GID*) (**B**). *PD*, protein digesta; *GD*, gastric digesta; and *GID*s, GI digesta. The different lowercase and uppercase letters and numbers indicated the differences in the mean values of the hydrolysate, *GD*s, and *GID*s, respectively (*p* < 0.05). Elution volume (*V*_e_) at 13.20, 18.10, and 20.77 mL was used to define the *M*_W_ distribution of the peptides, as detailed in Table 2.

The crude protein and fat contents of *P* were 83.87 ± 0.29% (d.b.) and 1.91 ± 0.33% (dw), respectively. The DH of the *P* hydrolysate increased rapidly within 6 h and then increased slowly afterward until reaching 35.36 ± 0.06% DH at 16 h (Figure 1A). Similar profile has been reported by others [14,18]. All the hydrolysates showed a similar pattern in *SEC*. Majority of peptides (~75%) in all samples exhibited apparent MW values ranging from 330–1250 Da (Table 2). This was the net result of the products from hydrolysis of large peptides and the conversion to smaller peptides. As the hydrolysis process was prolonged, the proportion of peptides having an MW < 330 Da increased in concomitant with a decrease of large peptides (MW > 1250). The smaller peptides were generated as DH increased during hydrolysis.

The protein and its hydrolysates were digested slightly by pepsin but significantly degraded by pancreatin (Figure 1A). Hydrolysates from threadfin bream (*Nemipterus* spp.) surimi byproduct [1] and trout frame [15] were also largely digested by pancreatin, but, only slightly by pepsin. Pepsin has a high specificity towards aromatic or Leu residues in both P_1_ and P_1′_ [41], resulting in a relatively low degree of digestion. In contrast, pancreatin is a mixture of endopeptidases and exopeptidases, which possess a broad specificity [26]. The protein showed the highest digestibility (*p* < 0.05, Figure 1A), while the hydrolysates showed a lower digestibility; in particular, the hydrolysates hydrolyzed for the extended hydrolysis time (>6 h) exhibited the lowest digestibility (*p* > 0.05, Figure 1A). Larger peptides were digested easily by the digestive enzymes, but shorter peptides are digested to a lesser extent due to the limited peptide bonds. All the digesta exhibited similar SEC patterns (Figure 1B) and MW distributions (*p* > 0.05, Table 2). Approximately 55% of the peptides in the digesta showed an apparent MW < 330 Da. Therefore, the protein and hydrolysates were digested to yield similar sizes of peptides in their digesta, and a majority of peptides exhibiting apparent MW < 330 Da. Thus, protein and its hydrolysate would eventually yield peptides with similar sizes upon GI digestion.

### 3.3. Chemical Antioxidant Activities

**Figure 2:**
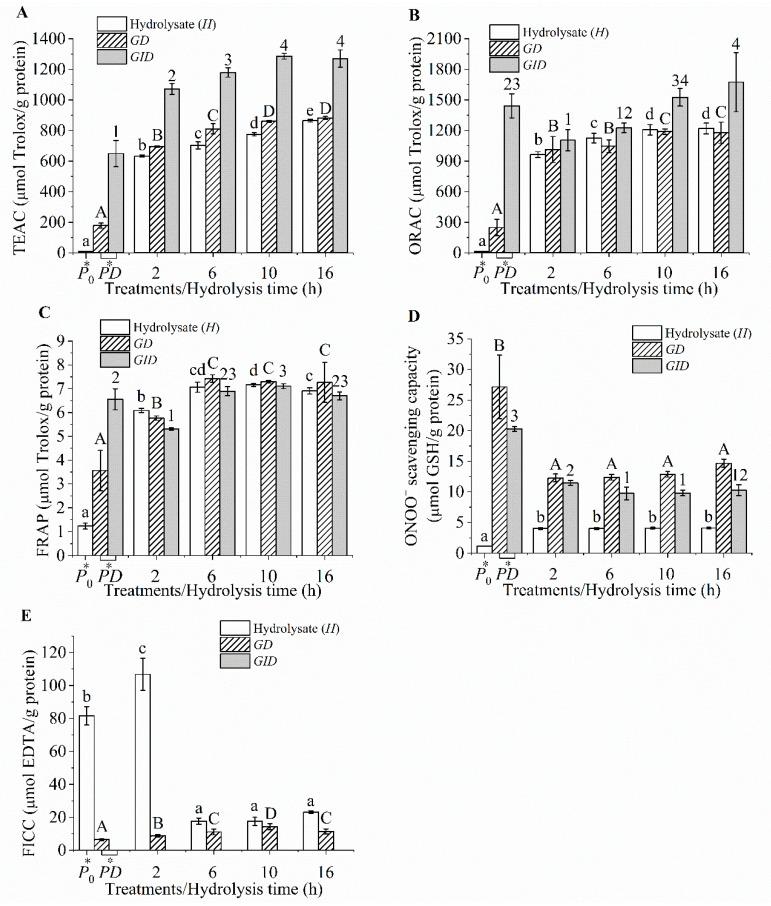
Chemical antioxidant activities of the hydrolysates and their digesta, Trolox equivalent antioxidant capacity (TEAC) (**A**), oxygen radical absorbance capacity (ORAC) (**B**), ferric reducing antioxidant power (FRAP) (**C**), ${\mathrm{ONOO}}^{-}$ scavenging capacity (**D**), and Fe^2^^+^ chelating capacity (FICC) (**E**). *P*_0_, heated protein; *PD*, protein digesta; *GD*, gastric digesta; and *GID*, GI digesta. The different lowercase and uppercase letters and numbers indicated the differences in the mean values of the hydrolysate, *GD*s, and *GID*s, respectively (*p* < 0.05).

The tilapia protein hydrolysates and their digesta exhibited various chemical antioxidant potentials (Figure 2A–E). High antioxidant values were observed for the TEAC and ORAC compared with those of the FRAP and ${\mathrm{ONOO}}^{-}$ scavenging capacity. The tilapia protein hydrolysates hydrolyzed by Flavourzyme, Protamex, and papain [39], as well as hydrolysates derived from fish skin gelatin and surimi byproduct [1,42], also exhibited a FRAP value that was lower than the TEAC value. The ORAC assay monitors the peroxyl radical (ROO•) scavenging activity via the hydrogen atom transfer (HAT) mechanism [43]. The FRAP and ${\mathrm{ONOO}}^{-}$ scavenging assays are related to the electron transfer (ET) mechanism [37,43]. The TEAC is a mixed-mode (ET/HAT) assay [43]. Therefore, the high TEAC and ORAC in the tilapia protein hydrolysate and digesta indicated that HAT might be the predominant mechanism responsible for the ability of tilapia protein-derived peptides to scavenge free radicals. The majority of amino acid residues are protonated at pH values of 3.6 (FRAP assay) and 7.4 (TEAC, ORAC, and ${\mathrm{ONOO}}^{-}$ assays). These protonated residues have the potential to donate a hydrogen atom and transfer the excess electron [44]. Therefore, it is likely that HAT contributed to the free radical scavenging capacity of the peptides.

In addition, thiol containing compounds such as Cys, GSH, and Cys-containing tripeptides were the effective ${\mathrm{ONOO}}^{-}$ scavengers [2,37,45], but tripeptides containing His and Tyr showed a weak ${\mathrm{ONOO}}^{-}$ scavenging activity [2]. Meanwhile, a lack of Cys (0.95% or less) has been reported in tilapia proteins [39,46]. Therefore, the weak ${\mathrm{ONOO}}^{-}$ scavenging ability of tilapia protein hydrolysates could also be attributed to a lack of Cys (˂30 μmol GSH equivalents/g protein).

The *H*_2_ exhibiting a DH of 20.97% showed a Fe^2^^+^ chelating capacity of 106.77 μmol EDTA equivalents/g protein, but low values (˂30 μmol) were observed for the hydrolysates at extended hydrolysis times (>2 h) and all the digesta (Figure 2E). The tilapia protein hydrolysates with DH values ranging from 10% to 40% also showed a negligible FICC (˂3 μmol EDTA equivalents/g solid) [39]. The gelatin hydrolysates obtained from farmed giant catfish skin (~10% DH) exhibited approximately 170 μmol EDTA equivalents/g sample [14]. Hydrolysates with higher DH values and their digesta did not possess a good metal chelating capacity because they contained shorter peptides with few negatively charged amino acid residues. Therefore, our results implied that HAT might be a predominant mechanism of peptides in hydrolysates and their digesta, which contributed to the antioxidant activity, while electron transfer and metal chelating were less important. The TEAC and ORAC appeared to be important assays for monitoring the antioxidant activities of the peptides derived from tilapia protein.

The TEAC and ORAC of the hydrolysate increased with increasing the hydrolysis time (Figure 2A,B). In addition, these values drastically increased after pancreatin digestion (*p* < 0.05). Smaller peptides possessed a better free radical scavenging capacity, and similar results have also been reported by others [4,18]. The shorter peptide is preferred to react with water-soluble ${\mathrm{ABTS}}^{\text{•}+}$ and ROO• radicals, leading to higher TEAC and ORAC values, respectively [47]. The lower scavenging power of longer peptides is attributed to an increased repulsion of bulky peptides [4]. Moreover, amino acid residues with an antioxidative potential might be hidden inside steric structures as inactive forms. These hidden amino acid residues are exposed during hydrolysis, leading to an improvement in the activity [48]. Thus, the TEAC of the hydrolysate increased continually and reached the highest in the *H*_16_ sample and further increased after GI digestion. After all the active residues were exposed upon proteolysis, the antioxidant activities became constant, as observed in the digesta of *H*_10_ and *H*_16_. It should be noted that the activities of the *H*_10_ and *H*_16_ digesta were comparable, although the parent hydrolysates of the latter showed higher activities (*p* < 0.05). Therefore, it might not be necessary to search for the hydrolysate with the highest antioxidant activity, but rather the activities of the digesta should be considered when optimizing the production of antioxidative hydrolysates. In addition, the protein digesta showed lower TEAC and ORAC values than did the hydrolysate digesta (Figure 2A,B). Since both digesta appeared to have similar sizes of peptides (Table 1), such differences in antioxidant activity could be mainly attributed from varied peptide sequences.

Pearson correlation analysis showed that the TEAC and ORAC of the hydrolysate were significantly correlated with the DH (*r* = 0.95 and 0.91, respectively). In addition, the TEAC and ORAC of the digesta were less correlated with the values obtained from the hydrolysates (*r* = 0.80–0.85). Our results indicated that the chemical antioxidant activities of the hydrolysate could not be used to predict the activities of its digesta. This is likely due to the differences in the size and sequence of antioxidant peptide sequences of the hydrolysates and their digesta. Hydrolysis time of 10 h (*H*_10_) rather than 16 h (*H*_16_) would be the optimal as it yielded hydrolysate with the highest antioxidant activity upon GI digestion. Thus, the activity of the hydrolysate digesta should be considered as one of the criteria to optimize production of antioxidative protein hydrolysate.

### 3.4. Cellular Antioxidant Activities

**Figure 3:**
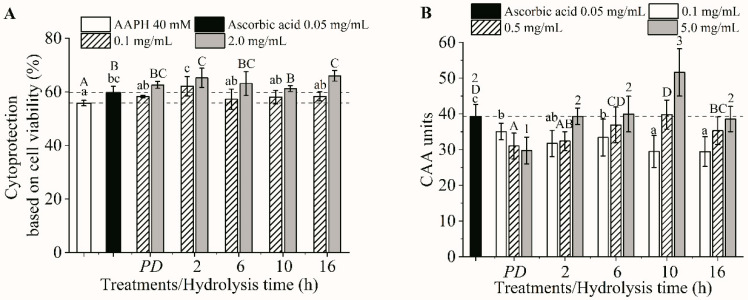
Cytoprotection at 0.1–2.0 mg/mL (**A**) and intracellular ROS scavenging capacity at 0.1–5.0 mg/mL (**B**) for all the digesta. *PD*, protein digesta. The different lowercase and uppercase letters and numbers indicated the differences in the mean values of the digesta at each concentration (*p* < 0.05). AAPH, 2,2′-Azobis (2-methylpropionamidine) dihydrochloride.

All the digesta were noncytotoxic up to 5 mg/mL and exhibited a dose-dependent cytoprotection behavior and intracellular ROS scavenging activity, except for the protein digesta (*PD*), whose CAA value decreased as the concentration increased (Figure 3A,B). All the digesta with concentrations of 0.1 mg/mL exhibited a cytoprotection capability that was comparable to that of 0.05 mg/mL ascorbic acid (*p* > 0.05), while the digesta of *H*_2_ was the most effective (*p* < 0.05, Figure 3A). However, the cytoprotection of all the digesta with concentrations of 2.0 mg/mL was comparable (*p* > 0.05). Based on the ROS scavenging activity, the digesta of *H*_10_ with a 0.5 mg/mL concentration revealed he highest activity comparable to the 0.05 mg/mL ascorbic acid (*p* > 0.05). In addition, the digesta of *H*_10_ showed the highest CAA unit at a concentration of 5 mg/mL (Figure 3B). Note that the protein digesta showed the lowest CAA unit (*p* < 0.05). These results indicated that the digesta of the protein and its hydrolysates possessed similar cytoprotective capacities, but the *H*_10_ digesta was the most effective ROS scavenger. It can be seen that the cytoprotective results did not agree with the intracellular ROS scavenging activity. This discrepancy has been previously reported [17,49]. The intracellular ROS scavenging capacity assay monitors the ability of the compound to remove intracellular ROS, while cytoprotection measured the cell viability, which is related to the ROS-induced oxidative stress that causes membrane damage and biomolecular dysfunction, as well as the mitochondrial dysfunction and/or DNA damage that causes apoptosis [49], and so on. Therefore, the same peptides might display various cytoprotection and ROS scavenging activity responses. The low ROS scavenging activity of the protein digesta, corresponding to the lowest TEAC and ORAC (Figure 2A,B). These results implied that protein digesta contained peptides with lower cellular antioxidant activity than did hydrolysate digesta. Different peptide sequences in the protein and hydrolysate digesta should be the main reasons contributing to the varied cellular antioxidant activities. Our results demonstrated that production of protein hydrolysate is an effective means to improve the antioxidant activity of tilapia proteins, but excessive hydrolysis (16 h) resulted in a reduction of CAA of hydrolysate digesta.

### 3.5. PCA

**Figure 4:**
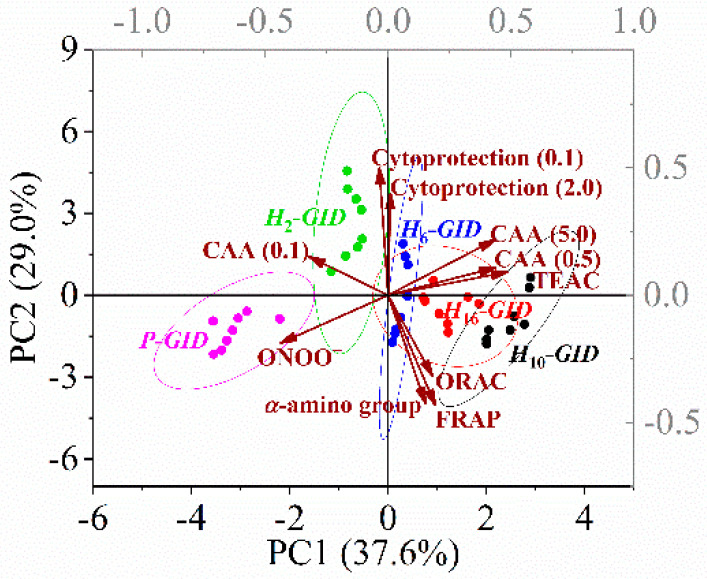
Principal component analysis (PCA) biplots (PC1 versus PC2) of the digesta of tilapia protein and its hydrolysates based on their *α*-amino group content and chemical and cellular antioxidant activities. Cytoprotection (0.1 or 2.0) and cellular antioxidant activity (CAA) (0.1, 0.5, or 5.0) indicated the activities of the digesta at concentrations specified in parentheses in mg/mL.

According to the chemical and cellular antioxidant activities of the digesta of the protein and its hydrolysates, almost 66% of the total variance can be explained by PC1 and PC2 (Figure 4). The protein digesta was clearly separated from the hydrolysate digesta with a high correlation to the ${\mathrm{ONOO}}^{-}$ scavenging activity. The digesta of *H*_10_ exhibited distinct characteristics of the TEAC and CAA (at 0.5 and 5 mg/mL). The tilapia protein hydrolyzed by Alcalase for 10 h appeared to show the optimal antioxidant activities upon in vitro GI digestion.

The loadings of the variables showed that PC1 was positively correlated with the TEAC and CAA (0.5 and 5.0 mg/mL) of the digesta. PC2 was positively correlated with the cytoprotection (at 0.1 and 2 mg/mL) but negatively correlated with the ORAC, FRAP, and *α*-amino group content of the digesta. The Pearson correlation showed that the TEAC of the digesta was positively correlated with its CAA at both 0.5 and 5.0 mg/mL, where *r* = 0.70 and 0.74, respectively. It was suggested that the TEAC could be used to predict the intracellular ROS scavenging capacity of peptides in the digesta. In contrast, the TEAC of the hydrolysates showed a lower Pearson correlation with the CAA (0.5 and 5.0 mg/mL) of the digesta, where *r* = 0.62 and 0.70, respectively. The chemical antioxidant activities of the hydrolysates also showed a weak correlation with those of their respective digesta, as discussed in Section 3.3. Therefore, TEAC of hydrolysate digesta showing the highest correlation with the CAA of digesta could be used to screen the antioxidant peptides derived from tilapia muscle proteins. Protein digesta was distinctively partitioned from the hydrolysate digesta with lower values of TEAC and CAA. Digesta of *H*_10_ showed high correlation with TEAC and CAA. Thus, the excessive hydrolysis (16 h) generated hydrolysate showing the highest antioxidant potential but did not yield the maximum capacity upon digestion. As activities of digesta are more closely related to health, the static in vitro GI digestion of hydrolysate should be used as one of the criteria for optimizing production of antioxidative protein hydrolysate.

## 4. Conclusions

The antioxidant activities of peptides derived from proteases used in the standard INFOGEST in vitro GI digestion should be systematically analyzed and subtracted from those of samples to better evaluate the true activities of the digesta. The antioxidant activities of the hydrolysate did not appear to reflect the activities of the digesta. The digesta of *H*_16_ exhibited lower antioxidant activities than did the *H*_10_ digesta although the hydrolysate of the former possessed higher activities. The protein digesta also showed the least antioxidant activities. Our results demonstrated that excessive hydrolysis of 16 h might not necessarily yield peptides with the highest activities upon in vitro GI digestion. The antioxidant activity of the hydrolysate digesta should be considered when optimizing the condition used for production of the protein hydrolysate with an antioxidant activity.

## Figures and Tables

**Table 1:** Alpha-amino group content and chemical antioxidant activities of enzyme blanks ^1^.

	Samples	*A*_0_	*Pep*-I	*Pep*-*Pan*-I	*A*_0_-*GID*	*H*_10_-*GID*^#^
Parameters						
*α*-Amino (mmol *L*-Leu)	Free	0.02 ± 0.00 ^a^	0.01 ± 0.00 ^a^	1.05 ± 0.01 ^b^	1.02 ± 0.02 ^b^	2.69 ± 0.09 ^c^
	Total	–	–	–	2.00 ± 0.15 ^a^	4.73 ± 0.12 ^b^
TEAC (μmol Trolox)		12.18 ± 0.98 ^a^	39.02 ± 0.00 ^b^	389.54 ± 0.55 ^c^	487.47 ± 0.65 ^d^	1004.83 ± 7.35 ^e^
FRAP (μmol Trolox)		0.11 ± 0.08 ^a^	0.03 ± 0.00 ^a^	1.94 ± 0.04 ^c^	1.78 ± 0.04 ^b^	4.64 ± 0.04 ^d^
FICC (μmol EDTA)		5.99 ± 0.40 ^a^	ND	7.33 ± 0.68 ^ab^	11.59 ± 2.11 ^c^	8.25 ± 0.35 ^b^
ORAC (μmol Trolox)		12.39 ± 1.09 ^a^	0.57 ± 0.03 ^a^	235.69 ± 58.40 ^b^	370.63 ± 10.35 ^c^	984.54 ± 34.18 ^d^
${\mathrm{ONOO}}^{-}$(μmol GSH)		ND	ND	0.59 ± 0.05 ^a^	1.06 ± 0.16 ^b^	3.95 ± 0.19 ^c^

^1^ The results were calculated as the sample that was brought to 25 mL. ^a^^–^^e^ Different lowercase letters in the same row indicated the differences in mean values (*p* < 0.05). ^#^ Without enzyme blank subtraction. ‘–’, the values were not determined; ND, the values were not detected; *A*_0_, thermally-inactivated Alcalase; *Pep*-I, blank of gastric (pepsin) digestion; *Pep*-*Pan*-I, blank of GI (pepsin and pancreatin) digestion; *A*_0_-*GID*, GI digesta of *A*_0_; and *H*_10_-*GID*, GI digesta of 10-h hydrolysate.

**Table 2:** Molecular weight (MW) distribution of protein hydrolysates, digesta, and enzyme blanks ^1^.

Sample	Percentage of Area under Chromatogram			
	>1250 Da	1250–330 Da	330–220 Da	<220 Da
Protein hydrolysates				
*H*_2_	12.29 ± 1.31 ^c^	77.49 ± 1.67 ^b^	10.00 ± 0.36 ^a^	0.22 ± 0.12 ^a^
*H*_6_	7.60 ± 0.68 ^b^	75.64 ± 1.88 ^ab^	15.59 ± 0.57 ^b^	1.17 ± 0.76 ^ab^
*H*_10_	4.46 ± 0.30 ^a^	75.55 ± 0.92 ^ab^	18.73 ± 0.70 ^c^	1.25 ± 0.39 ^ab^
*H*_16_	3.61 ± 0.54 ^a^	73.52 ± 0.34 ^a^	21.02 ± 0.75 ^d^	1.85 ± 0.15 ^b^
Digesta of protein hydrolysates				
*P*-*GID*	5.79 ± 0.30 ^A^	37.50 ± 0.27 ^BCD^	35.27 ± 0.37 ^BC^	21.44 ± 0.36 ^D^
*H*_2_-*GID*	6.13 ± 0.11 ^A^	37.74 ± 0.38 ^CD^	35.70 ± 0.32 ^BC^	20.43 ± 0.55 ^CD^
*H*_6_-*GID*	5.71 ± 0.21 ^A^	38.77 ± 0.61 ^D^	36.24 ± 0.16 ^BC^	19.28 ± 0.27 ^ABC^
*H*_10_-*GID*	5.76 ± 0.24 ^A^	37.59 ± 0.16 ^BCD^	36.61 ± 0.30 ^C^	20.04 ± 0.27 ^BC^
*H*_16_-*GID*	6.07 ± 0.45 ^A^	37.52 ± 0.26 ^BCD^	36.59 ± 0.46 ^C^	19.82 ± 0.24 ^BC^
Enzyme blanks				
*Pan*_0_	16.47 ± 0.34 ^D^	36.57 ± 0.49 ^BC^	28.90 ± 0.96 ^A^	18.06 ± 0.69 ^A^
*A*_0_-*Pep*_0_-*Pan*_0_	14.96 ± 1.08 ^C^	36.32 ± 0.11 ^B^	29.83 ± 1.40 ^A^	18.90 ± 0.42 ^AB^
*A*_0_-*GID*	8.99 ± 0.43 ^B^	26.87 ± 0.74 ^A^	34.57 ± 0.64 ^B^	29.56 ± 0.71 ^E^

^1^ MW at elution volume (*V*_e_) 13.20, 18.10 and 20.77 mL were calculated by formula LogMW = −0.649ln (*K*_av_) + 2.1656 R^2^ = 0.988, respectively, and used to define the MW distribution of peptides. ^a^^–^^d^ Different lowercase letters indicate differences in mean values among protein hydrolysates (*p* < 0.05). ^A^^–^^D^ Different uppercase letters indicate differences in mean values among digesta and enzyme blanks (*p* < 0.05). *A*_0_, *Pep*_0_, and *Pan*_0_ were referred to as thermally-inactivated Alcalase, pepsin, and pancreatin, respectively; *A*_0_-*Pep*_0_-*Pan*_0_, the mixture of *A*_0_, *Pep*_0_, and *Pan*_0_; and *A*_0_-*GID*, digesta of *A*_0_.
